# Dental caries: A complete changeover (Part II)-Changeover in the diagnosis and prognosis

**DOI:** 10.4103/0972-0707.57631

**Published:** 2009

**Authors:** Usha Carounanidy, R Sathyanarayanan

**Affiliations:** Department of Dentistry, Pondicherry Institute of Medical Sciences, Pondicherry, India; 1 Department of Conservative Dentistry and Endodontics, Bapuji Dental College and Hospital, Davangere, Karnataka, India

**Keywords:** Active caries, caries activity, cavitated caries, dental caries, detection, diagnosis, inactive caries, noncavitated caries, prediction, prognosis, risk assessment, sensitivity, specificity

## Abstract

Realization that dental caries is a reversible, dynamic biochemical event at a micron level has changed the way the profession recognizes the caries disease and the caries lesion. The diagnosis of dental caries poses challenges due to the complex interaction of multiple endogenous causal factors. The most appropriate diagnostic aid for this purpose is the risk model of caries risk assessment. The analyses of the biological determinants provide clues to the dominant causal factor. The detection of a carious lesion has undergone a rigorous revision and revolution in order to identify the earliest mineral change so that it can be controlled without resorting to invasive management options. Apart from detection, it became mandatory to assess the extent of the lesion (noncavitated/cavitated), assess the activity status of the lesion (active/arrested), monitor the lesion progress (progression/regression over a period of time), and finally to predict the prognosis of the lesion as well as the disease. The prognosis of the disease can be best assessed by analyzing the predictor factors in caries risk assessment. The ultimate objective of such a meticulous and methodical approach aids in devising a tailor-made treatment plan, using preventing measures precisely and restorative measures minimally. This ensures the best oral health outcome of the patient.

## INTRODUCTION

The preceding part of this series had focused on the changeovers in dental caries definition and etiopathogenesis.[1] They are recapitulated as follows:

1. Dental caries is a disease with multiple causal factors, called as determinants and confounders, manifesting itself on the tooth structure as a carious lesion. Such manifestations may range from microscopic to macroscopic changes in the tooth.
2. The underlying causal process has been attributed to disturbances in two oral homeostases: (a) disturbance in the mineral homeostasis between the tooth and the oral fluid and (b) disturbance in the microbial homeostasis in the biofilm.
3. The result is alternate cycles of mineral loss and gain in the hydroxyapatite crystal. These cycles are orchestrated by a set of factors called as the demineralizing factors and the remineralizing factors.

With this knowledge in the backdrop, the ensuing text shall unfold the changeovers in dental caries diagnosis and prognosis.

## DIAGNOSIS OF DENTAL CARIES DIASESE – THEN AND NOW

Diagnosis is “the recognition of a disease or a condition by its outward signs and symptoms”.[2] The diagnostic process in medical field is called as the hypothetico-deductive process, where data are collected methodically to zoom-in to the particular disease, from a list of differential diagnoses.[3] Devising a treatment to cure the patient from the disease and its associated symptoms is the end point of a diagnostic process in medicine.[4]

If dental caries is a disease and the lesion is the sign of the disease, then even a dental clinician should follow the same. But in reality, this process of elimination does not take place in the diagnosis of dental caries. A dental clinician is instantly and intuitively aware that certain unique demineralizations and destructions observed on the tooth are tell-tale signs of none other than a dental caries disease. Therefore, diagnosis is rarely about finding out what the patient has but does the tooth have caries.[5]

Generally, it is a common practice that on dental examination, if demineralizations or destructions are observed, at whatever stage, they are immediately labeled as “dental caries.” Probably, the labeling may extend a little further, to include the nature and extent of the lesion (e.g., DC class II MO). Once the lesion is labeled, a treatment plan is devised for that lesion (e.g., silver amalgam class II MO). Thus diagnosis and treatment of the disease shrink down to just labeling and treating the lesion. Due to the absence of important steps such as differential diagnosis and diagnosis, probably, the carious lesion takes the center stage eclipsing the importance of the caries disease. An inevitable query rises with this practice of using the “sixth sense” in caries diagnosis; has the dental profession ever bothered to diagnose and treat dental caries disease? The answer to this can be elucidated by revisiting the dental caries causal process.

## DENTAL CARIES – PROCESS OR PATHOLOGY?

**Figure 1:**
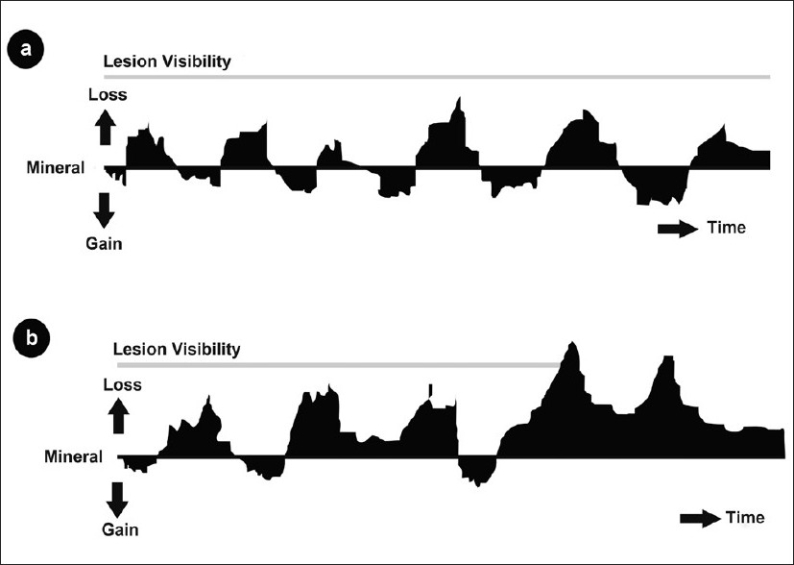
Illustration depicting the micro biochemical events occurring on the tooth- biofilm interface over time. (a) The mineral loss and gain balanced and lesion not visible (b) The mineral loss and gain not balanced and lesion is visible as white spot[6]

In the presence of the biofilm, every sucrose attack creates an acidic environ in the immediate vicinity of the tooth. This initiates a sequence of random biochemical reactions of mineral loss and mineral gain. As long as the dynamic equilibrium of the mineral content of the tooth-oral fluid and the microbial content of the biofilm is maintained, the entire sequence remains within the boundary of a physiological process, where loss and gain are equalized[6] [Figure 1]. Given the fact that the tooth is always cloaked under a blanket of the biofilm, this concept of dental caries being a physiological process is further reinforced. Thus dental caries is also thought to be a ubiquitous physiological process, ever present in the oral cavity.[7]

However, if the factor disturbing the homeostasis is strong, intense, and long lasting, then the demineralization persists and prevails. The mineral loss is now more than the gain, which becomes evident as structural changes in the tooth structure. Along a timeline, this shift in the balance leads to more changes in the tooth structure, which cannot be accepted as healthy, but should be considered as pathological. Thus the physiological dental caries process becomes a pathological dental caries disease.

## PROCESS TO PATHOLOGY CONTINUUM - A PROBLEM IN DENTAL CARIES

The transformation of dental caries from a physiological process to pathology is not a sudden cross-over, but a continuum over a period of time, affected by numerous variables. There is a lack of a definite boundary line between health and disease. This becomes the raison d'être for the inextricable confusion in the diagnosis of dental caries and even in the detection of its manifestation. The profession is perpetually burdened by the question “when is caries, caries.”[8] Extensive research is being spent to pinpoint the starting point of the disease process and the ending point of the physiological process, but in vain. Different studies choose different cut-off points in the continuum as a baseline. The criteria used by the epidemiological surveys are different from those used in clinical settings. Lucid frame lack in differentiating “sound” or “diseased” tooth results in vagueness of diagnosis and confusions in treatment decisions as well. Indecisions such as “to treat or not to treat,” and “to do less or to do more” hover the treatment plan, resulting in either overtreatment or undertreatment. The ultimate objective of an accurate diagnosis is to provide a wholesome treatment, and to err in this objective is indubitably undesirable.

**Figure 2:**
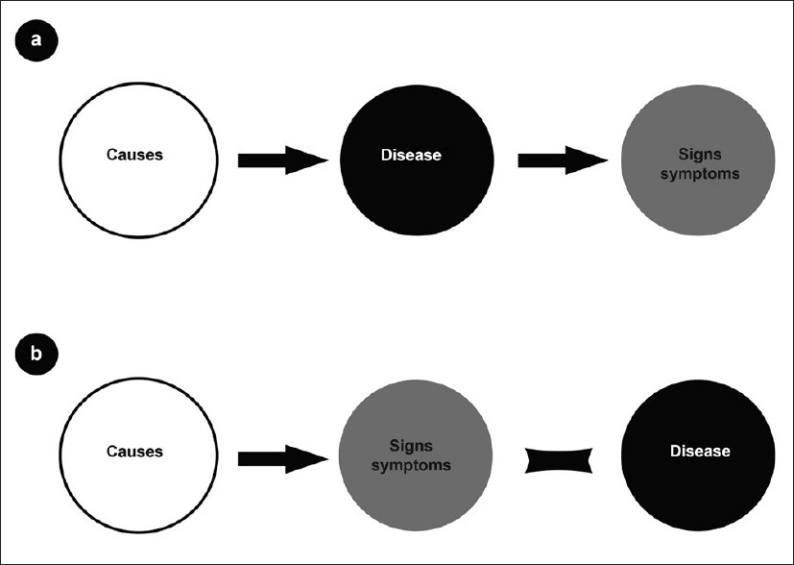
Illustration depicting a new concept on Dental caries (a) Essentialistic concept: The causes result in a disease that manifest as signs and symptoms. (b) Nominalistic concept: The disease name is no more than a label given to certain characteristics of the signs[10]

Therefore, in order to achieve this goal of best management options for the patients, a shift in disease paradigm has been suggested.[9] The concept of understanding caries as the disease and lesion as the manifestation is based on a philosophy called as essentialism. This concept seems to fail in bringing out a definite baseline or gold standard, “caries truth.” It is clearly evident from the above discussion that the lacuna is simply due to a complex caries process. Nominalism, a philosophy that is directly opposite to essentialism, is being recommended as the need of the hour, to circumvent this problem. In the nominalistic concept, the ultimate objective is to arrive at the best treatment outcome for the patient, not wasting time in finding out the invisible disease or the indefinable caries truth. According to this concept, the disease name is just a convenient way of labeling a set characteristics defined as the lesion. Here it is implied that “the disease and the signs are the same; in other words, the disease does not exist; only a causal process exists that results in clinical signs and symptoms”[10] [Figure 2]. Thus a dental clinician uses a method of pattern recognition through a nonanalytical thinking and match the signs present on the tooth with the previously stored caries scripts.[5] Caries scripts are a mental inventory created in the clinician's mind based on the experience of numerous previous presentations of the carious lesion. The signs contained in the script are usually visual and less frequently tactile. Once a clinician observes a particular sign on the tooth, automatically a mental matching is done with the caries scripts. These scripts are inseparably linked to the treatment. Thus, an intervention ensues immediately. The length and spectrum of this inventory are influenced by the knowledge, experience, and attitude of the clinician, and continue to be updated with the changing presentation of carious lesions. Stripped off the scientific details, caries scripts are similar to the data stored in a computer. This concept apparently is no more different from yesteryear's “labeling” of the lesion. The science of cariology might be making an about turn in the concept of diagnosis, from then to now! Evidence to support his new thinking is much awaited.

However, diagnosis has another meaning: “the analysis of the underlying physiological/biochemical cause(s) of a disease or condition.”[2] Instead of asking when and where a process ends and a disease starts, or asking if it is possible to identify such a line of control, it might be prudent as well as imperative to ask why the disease started. Simply stated, identifying the cause for such a pathological shift is also a part of the diagnostic process of a complex disease like dental caries. Generally, once the cause is identified, the treatment includes a strategy for its elimination. However, dental caries is a product of interaction of multiple causative factors that are not exogenous, but endogenous, so it is not possible to eliminate them in a true sense. Nevertheless, any or all of these endogenous factors can gain a pathogenic status due to differing reasons. They can exert an unfavorable effect on the oral homeostasis, thus tipping the balance toward disease. If such factor/s can be identified and rectified, not eliminated, then the caries machinery can be shifted to the reverse gear, from pathology to physiology. This particular dimension of the diagnostic process in dental caries is best accomplished by caries risk assessment (CRA).

### Caries risk assessment

Risk assessment primarily intends to identify the individuals who are prone to the disease. But caries risk assessment can be comprehended as two models, namely, risk model and prediction model. A risk model identifies one or more causative factors of the dental caries disease. The prediction model identifies the patient who is at high risk for the disease. Generally, three approaches have been proposed for risk assessment, such as past caries experience, socioeconomic factors, and biological factors. The past caries experience and the socioeconomic factors form part of the predictor model and the biological factors, such as diet, saliva, and the microbes, are used both in risk and predictor models.[11] In other words, the confounders at the periphery of the causal model are of prognostic value and the determinants in the center are of diagnostic as well as prognostic value.

In the following text, only the risk model associated with diagnosis will be discussed. The predictor model of CRA will be dealt under the section of prognosis of dental caries.

### CRA: The diagnostic aspect

The causal factors, in the Key's circle, are depicted in the form of four intersecting circles of a uniform dimension. At a glance, it might be interpreted that all the four factors have to be of equal strength and severity to cause dental caries, which is not true always. Assuming that all the causal parameters are responsible for the precipitation of the disease and then developing a treatment strategy that is all-inclusive is obviously a waste of resource. If two caries-inducing factors have different magnitudes, the end product is the same, i.e., dental caries. For instance 2 sucrose factors × 10 microbial factors = 10 sucrose factor × 2 microbial factors = dental caries.[12] The pathogenicity of even an isolated factor can tip the balance toward disease. For instance, in a thick, long-standing biofilm (a poor oral hygiene), acidity is retained for a long time, as the buffering capacity of the oral fluid might not be able to diffuse through such tenacity. In the same way, a sucrose attack that is persistent (a sweet tooth!) does not provide enough time for effective buffering. Once such dominant factor(s) is spotted through risk analysis, then a treatment plan can be tailor-made specifically, targeting the specific cause. Such a target-oriented treatment plan optimizes the patient compliance and his or her health outcome, with well-spent resources. In a situation where the biofilm factor is dominant, there oral hygiene measures take the top priority in the treatment strategy and in the situation where the sugar factor is dominant, and then the dietary counseling is the main frame of action. The other preventive measures trail as secondary strategies.

The robust literature is available on the methodology for CRA.[13–17] The data that are obtained through risk assessment can be qualitative or quantitative. The qualitative data are obtained from history, clinical examination, and dietary analysis, whereas the quantitative data are obtained from the salivary and the microbial analysis. Most of the details obtained from history and clinical examination relate to the status and prognosis of caries. Therefore only the dietary, salivary, and microbial analyses that are essential for the diagnostic process shall be dealt here.

Dietary analysis is the most subjective of all, as it involves patients' attitude, motivation, cooperation, and honesty. It is aimed at finding out the cariogenicity and the nutritive value of the diet consumed by the patient. The frequency of consumption of cariogenic diet is also of vital concern. The commonly used methods are dietary history, 24-h recall, dietary record, and food frequency questionnaires.[18,19] A 3–7 days dietary chart might provide a wholesome picture [Table 1]. The patient is educated and motivated to enter in the chart everything that he/she consumes from morning till bedtime. This should also include the medications, chewing gums, and the cough lozenges. Later it is analyzed by the dentist-patient duo for the frequency and quantity of frank or occult sugar intake. It is very imperative that the dentists update their knowledge on the nutrition facts and the cariogenic potential of various foods.

Salivary analysis is done to assess four parameters, such as flow rate, buffering capacity, pH, and viscosity. Both stimulated and unstimulated saliva can be used for this purpose.

Salivary flow rate is the most important clinical parameter affecting dental caries susceptibility.[20] With a reduced quantity of saliva, the oral clearance of the microorganisms and the food remnants is impaired. Consequently, the pH and the buffering capacity also reduce. This acidic environ results in the growth of the aciduric cariogenic organisms. The calcium and phosphate content also tends to drop down with reduced saliva, and thus the remineralization capacity is compromised. Though an unstimulated salivary flow is the ideal indicator of a low salivary flow rate,[21] it is very difficult to obtain the resting saliva in a state of consciousness. Thus it is a routine procedure to collect the stimulated saliva for evaluating the flow rate. A stimulated salivary flow less than 0.7 ml/min and an unstimulated salivary flow less than 0.1 ml/min are considered as a low flow rate.

**Table 1:** A 3-day diet chart

Diet	Day 1		Day 2		Day 3	
			
	Time	Item/quantity	Time	Item/quantity	Time	Item/quantity
**Before breakfast**						
Breakfast						
Before Lunch						
Lunch						
Evening						
Before dinner						
Dinner						
After dinner						

Any medications:

The unstimulated saliva is used to test the pH because the resting pH is the correct indicator of caries occurrence. With an increased flow, the pH can shift toward reduced acidity. The buffering capacity of the saliva is its ability to reduce the acidity. This can be measured by the buffer effect.[22] The buffer effect is assessed by mixing a fixed amount of acid to the fixed amount of saliva and checking the pH value. There are two buffering systems in the saliva that are critical in neutralizing the acidity created by a biofilm-sucrose interaction. They are the phosphate system and the bicarbonate system. The phosphates are the main buffering agent in unstimulated saliva and the bicarbonates are the prime ones in stimulated saliva. The bicarbonate system is more important in contributing to dental caries; therefore, stimulated saliva is the right sample to assess this. The final pH value ≤ 4 denotes a low buffering capacity of the saliva.

The function of minor salivary glands is assessed by testing the hydration capacity. Hydration is directly related to the accumulation and growth of the biofilm. The examination mirror sticks onto the mucosa in a dry mouth. Viscous, ropy saliva reflects less water content and thus affects the oral clearance rate. Normally, the ability to string out for saliva, when lifted with a mouth mirror, is just 2.5 cm.

Microbial analysis is done by culturing the salivary sample to assess the growth of S. mutans streptococci and the lactobacilli. The acceptable level of both organisms in the saliva is less than 1 million colony forming units (CFU). Laboratory culturing techniques are used to grow both organisms in their respective culture medium. The culture medium used for lactobacillus growth is Rogosa SL-agar and the one used for the growth of mutans streptococci is MSB-agar. Commercially available kits provide a plate, which contains culture media for the both organisms on either side. The plate is then incubated in the laboratory. Currently, chair-side assessment methods are commercially available that are based on a species-specific monoclonal antibody response, where the results are obtained within 30 min. An increased mutans streptococci level is usually evident in the presence of more incipient lesions. This is because of the ability of these organisms to adhere to the tooth by the extracellular polysaccharides, rendering them responsible for the initiation of the lesion. The lactobacilli require retentive areas to proliferate and hence, a high count is usually associated with the presence of frank cavitations.

Though the above-mentioned analysis is performed individually, it should be remembered that the data obtained should be evaluated collectively [Table 2]. It is not advisable to base the diagnostic decisions on a single result. The overlapping of the influence of one factor over the other should not be overlooked for devising a correct treatment plan. For instance, a high count of lactobacilli, even after the restoration of a frank lesion, correlates with a high intake of carbohydrate in the diet. In such an instance, both dietary factor and microbial factor have to be addressed simultaneously.

In an attempt to reduce the subjectivity of the data obtained through the risk analysis, quantification is attempted in a computerized analysis. This is called as the cariogram, where the factors are weighted and projected as a pie diagram. The weighted factors are depicted as sectors in the pie, with different color codes, and the remaining sector indicates the “chance to avoid cavities.”[11] A traffic light matrix (TLM model) risk assessment model[23] uses color codes such as red, orange, and green, to denote specific threshold values for the data obtained in the analysis. The objective is to alert the clinician regarding the current risk status. The model is designed to keep the visual interpretation simple and easily communicable to the patient.

## DETECTION OF CARIOUS LESION – THEN AND NOW

The objectives of clinical examination of the oral cavity are dual:

1. Examination for carious lesion
2. Examination of carious lesion

**Table 2:** The average normal values and risk values of the risk factors in CRA

Biological risk factors	Normal range values	Risk values
Stimulated salivary flow	1.5 ml/min	≤ 0.5–0.7 ml/min
Unstimulated salivary flow	0.3 ml/min	≤ 0.1 ml/min
pH of resting saliva	6.7–7.4	≤ 5.5
Buffering capacity and the final pH	5–7	≤4
S. *mutans* count	<105 CFU	>106 CFU
Lactobacilli count	<104 CFU	>105 CFU

CRA: Caries risk assessment, CFU:Colony forming units

### Examination for carious lesion

In the process of examination for caries, it is appropriate to use the term, detection, which means discerning something hidden or subtle.[24] The necessity to detect lesions at the earliest is essential in the realm of current cariology because, if detected early, these lesions can be remineralized using noninterventional or preventive therapy. In addition, the wide use of fluorides has rendered the superficial enamel resistant to decay; but the decay can progress through a defective fissure on the occlusal surface resulting in occult caries.[25]

A good diagnostic aid should express most, if not the whole truth, regarding the presence or absence of the disease and its related sign. An aid that is sensitive enough to produce maximum of true positive results and specific enough to produce maximum of true negative results, keeping the false negatives and false positives to the minimum are considered valid aids/correct aids. But the correct interpretation of the results is solely dependent on certain attributes of the clinician, namely, observation, knowledge, and discipline. So a diagnostic aid should also be designed in such a way that it culminates in reliable/reproducible results, with least inter-/intraobserver variation. In addition, an ideal lesion detection aid should be able to provide results with the same accuracy, in detecting signs of varying severity (e.g., noncavitated as well as cavitated) on various surfaces (e.g., occlusal, proximal, and root surface).

**Figure 3:**
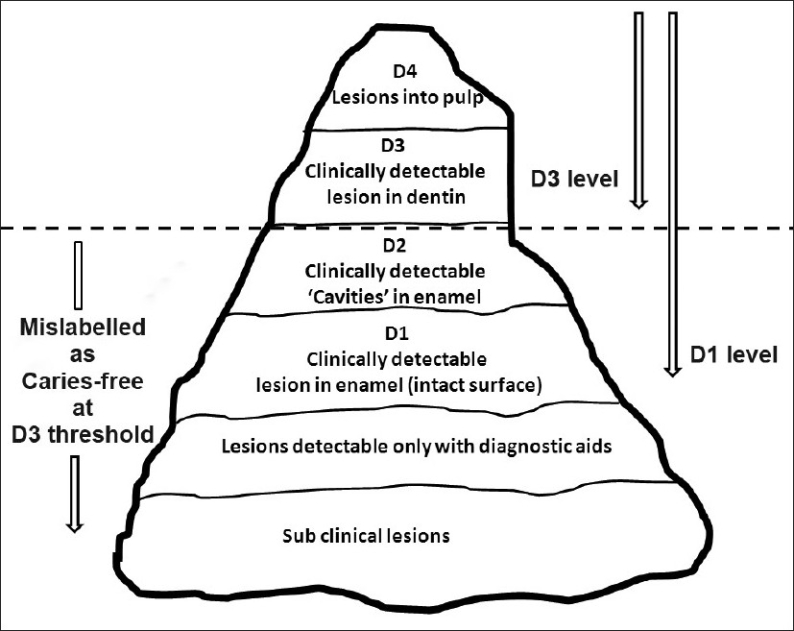
Illustration depicting a ‘Caries Iceberg’, showing various diagnostic thresholds[26]

Such a diagnostic aid for caries detection, till today, remains an evasive one. The main factor responsible is the intangible question, “how early is early?” This question stems from the indefinable caries process continuum; therefore, the answer is inexplicit as well. Every diagnostic aid should have a scale of measurement that is based on a diagnostic threshold. A diagnostic threshold determines what is recorded as “diseased” or “sound”[26] [Figure 3]. Unfortunately, in carious lesion detection, the scale of measurement differs with clinicians, researchers, techniques, and gadgets because a baseline or a gold standard is absent, as was discussed earlier. The end result is conflicting data on the sensitivity and specificity of various aids. The absence of such a stable caries diagnostic threshold, in addition, has profound effects on possible negligence of early pathological demineralization or on overindulgence of early physiological demineralization. To circumvent these problems, the current trend in caries diagnostics is focused on the following:

1. Formulation of stringent diagnostic criteria that is realistic and useful for the treatment decision.
2. Concurrent evolution of the technical gadgets, aiming at lowering the diagnostic threshold more and more, in order to detect the earliest mineral loss.
3. Revolutionary progress in quantifying the test results, in order to reduce inter- and intraobserver variation that is related to the qualitative assessment.
4. Shift from a dichotomous, nominal and ordinal scale, to a numerical scale that helps in monitoring the progress of the lesion over time.

### Traditional diagnostic aids – The inception

Seeing and touching are dual aspects of cognitive perceptions. Naturally, visual and visual–tactile examinations have been the most commonly used diagnostic aids in caries examination since many years. Visual inspection that was used previously lacked definite diagnostic criteria to detect an incipient lesion. Though it proved to be good in detecting cavitated lesions, it provided less sensitive results with regard to noncavitated fissure caries, and occult occlusal and approximal lesions.[27]

A probe or an explorer was used along with the visual aid in order to fulfill this lacuna. However, studies show that such a beneficial output was not evident.[28] In addition, the undesirable conversion of noncavitated lesions to cavitated lesions occurred with aggressive probing preventing the institution of preventive measures.[29] Without an input of concrete diagnostic criteria, namely, how catchy is a catch, how sticky is sticky, and how gentle is gentle, this aid gradually became invalid. However, a gulf still exists between two continents regarding its use.

The introduction of roentgen rays opened up the third eye for the dental profession. In dental caries examination, it proved to be a vital tool in detecting the occult proximal lesions. It was also widely used to assess the depth of the lesion. Bite-wing radiography has been in practice since 1925, and it is preferred to the periapical radiographs mainly because of the superior imaging geometry for visualizing caries.[30] But the validity of radiography in detecting incipient enamel lesions in both occlusal and approximal surfaces is low. Nevertheless, it is very specific in detecting the dentinal lesions in both surfaces.[31] This can be related to the fact that 40–60% of tooth decalcification is required to produce the radiographic image.[32] The depth of the lesion is underestimated most often. A wide range of intraobserver variation has been documented in interpreting the radiographic image that is strongly related to the technical manipulation of conventional films and the rays. Therefore, more false positive results were encountered. Ethical issues regarding the radiation exposure is an added handicap. These disadvantages underscore the importance of prescribing or interpreting a radiograph in a clinical setting, along with visual or visual–tactile examination.

Yet another aid that has been in use for the detection of occult caries is the fiber-optic transillumination test (FOTI). This device is based on the principle of light scattering. As light is scattered more in a carious tissue than in a noncarious tissue, it is observed as a dark shadow against a light background. Initially, it was used for the detection of approximal lesions mainly in the anterior teeth. But later its use was extended to detect proximal lesions in the posterior teeth as well. The hidden dentinal caries in the occlusal aspect is well detected with a three-dimensional view indicating the volume of caries. Compared with the bite-wing, it has been reported to be equally specific, but less sensitive.[33] However, due to the ease of operation, low cost, and absence of ionic radiation exposure, it is suggested to be better than its contemporary. But it should be noted that the interpretation is based on shadows that is akin to a radiographic interpretation, which can be misleading.

Probe, bite-wing radiograph, and FOTI reported reduced specificity when used alone, whereas, visual examination reported poor sensitivity. Therefore, they have been used in combinations, to improve sensitivity without compromising much on specificity. Nevertheless, from raw versions, all these traditional aids have undergone some internal restructuring over these years, with a simple objective of becoming more valid and reliable.

### Traditional aids – The progression

A major drawback in visual examination was the use of varied diagnostic criteria by various authors. An exhaustive review regarding this concluded emphasizing on the need for one criteria system.[34] Thus emerged the ICDAS (International Caries Detection and Assessment System).[35,36] This is a formulation of unambiguous explicit criteria, for clinical visual inspection, based on the already existing best evidence. The objective is to lead to better quality information to inform decisions about diagnosis, prognosis, and clinical management of caries at both individual and public health's level. Coronal caries (pits and fissures, mesial-distal, and buccal-lingual), root caries, and caries associated with restorations (CARS) are individual sections with individual criteria that are dealt under ICDAS. Basically a two-digit coding system is used to describe the detection and the status of the lesion/restoration. The first digit classifies each tooth surface on its restoration status. The second digit records the caries severity of a tooth surface. The caries severity assessment is based on the color and surface texture. Important prerequisites for such an examination are compressed air, and cleaning the biofilm from the tooth surface with the help of a round-ended probe and with prophylaxis paste. The criteria and the coding are elaborated in www.ICDAS.org. Numerous studies are on to assess the validity and reliability of this system. With the current evidence, the system seems to meet the requirements of a good detection aid. The sensitivity and specificity values have been improved for the detection at D1 (enamel and dentinal lesions) and D3 (dentinal lesions only) level.[37]

After a long debate of “to probe or not to probe,” probing has resurged now as a valuable adjunct to visual examination, but with a different purpose and well-defined criteria. Today's probing is limited to the removal of plaque from the surface of an incipient lesion, thus enhancing visibility. In addition, it is used to assess the surface texture of a lesion, an attribute that implies the activity of the lesion. The mode of usage changed too. Blunt probe, manipulated at a 20–40° angle to the surface, is being recommended against a sharp probe acting perpendicular to the tooth surface.[38]

Radiographic detection has evolved to digital radiography, with the hope of shedding all the pitfalls of the conventional radiography. But studies reported digital bite-wing radiography, in terms of the diagnostic aid parameters, to be only as accurate as the conventional ones, not any better.[39] Despite this, with less exposure radiation, quick imaging, and easy storage, direct digital radiography seems to score above the conventional method. In addition to these advantages, the possibility of image enhancement in digitization is currently very attractive. Studies do show that the sensitivity of enhanced imaging is superior to that of conventional films, but at the cost of lowered specificity.[40] Automated caries detection by computer software, digital subtraction radiography, and tuned aperture computed tomography (TACT) are the other diagnostic aids that have emerged in the same league.

Digitization has been adopted in FOTI also, to improve the sensitivity. This device is called the digital imaging fiber-optic transillumination (DIFOTI). The device works on the same principle of light scattering, but the human eyes are replaced by CCD intraoral camera to capture the image and instantly project in the monitor. As discussed for direct digital radiography, image enhancement, storage, and comparison between images are its main advantages. The sensitivity has been reported to be better than bite-wing radiography, but strong evidence is still lacking for this device.[41]

### Novel detection aids – The inception and the future

Novel detection aids evolved with an aim to revolutionize the caries detection at a very incipient stage, by further lowering the diagnostic threshold. These devices utilize the subtle changes that happen in the physical properties of a demineralized tooth structure. Optical, electrical, and thermal properties of a demineralized tooth differ from the sound tooth structure. Observations of the interaction of energy that is applied to the tooth structure or observations of energy that is emitted from the tooth are the basic platforms on which these hi-tech gadgets operate.[42] Gadgets that use electrical, visible light, UV light, infrared, tetrahertz, and sound waves are all being proposed for early detection. Optics-based gadgets such as multiphoton imaging, infrared fluorescence, optical coherence tomography, and tetrahertz imaging, and ultrasound devices that use sound waves and infrared thermography that uses thermal waves are all in the developmental stage. The most widely used optical devices are based on light scattering and laser/light-induced fluorescence and the electrical devices are based on electrical conductance and electrical impedance.

Fluorescence is a phenomenon where the wavelength of the light coming from the source loses energy to the surrounding tissue, and thus is converted to a larger wavelength of different color when it travels back toward the observer. Enamel and dentin have a certain fluorescence that is called as autofluorescence. This is attributed to some chromophores present in the dental structure. Carious lesions as well as the biofilm fluoresces on incident light. This is attributed to protoporphyrin, which is a bacterial break-down product. The difference in the fluorescing capacity of the sound tooth and the carious lesion can be recorded or observed. Two gadgets are available in this concept: quantitative light-induced fluorescence (QLF) and the Diagnodent (KaVo).[43,44]

Diagnodent uses a red light at 655 nm wavelength, emitted from a laser source. Caries-induced changes fluoresce in red color due to the presence of the bacterial by-product. So, logically the Diagnodent device can detect the deep lesions with bacteria and not the superficial lesions where bacterial fluorophores are absent. The more intense the fluorescence, the more is the destruction. The intensity is displayed in a continuous numerical scale from 0 to 99. A recent modification of Diagnodent is the Diagnodent pen, which has a tip of 0.4 mm; thus it facilitates easy placement in the approximal areas for the detection of an incipient proximal lesion. Systematic reviews are available that elaborate on the performance of Diagnodent on the occlusal and proximal lesions.[45,46] The sensitivity of the gadget is good in detecting occlusal dentinal lesions, but the specificity is generally lower than visual inspection. Evidence is very less for its efficacy in detecting the proximal lesions, in spite of the modification in the tip. Diagnodent has a tendency to provide errors in the presence of stains, biofilm, and fillings, but it has the advantage of monitoring the progression/regression of the incipient lesion over time.

Quantitative laser/light-induced fluorescence (QLF) is a device that works on the same principle of fluorescence of the tooth and the carious lesion, but uses a light source emitting a blue light in a shorter wavelength of 488 nm. The fluorescence emitted back in the yellow region is observed through a yellow high-pass filter to filter out all the reflected and back-scattered light. The yellow fluorescence is attributed to the proteinic chromophores that are cross-links between the chains of structural proteins. Light scattering is more in the demineralized enamel; thus absorption, and hence fluorescence are also less. In addition, the scattered light acts as barrier for the light to reach the underlying healthy tooth and also for the fluorescent light from the tooth to reach the observer. The net result is a less penetration depth and visualization of the enamel lesion as a dark spot against a light green fluorescent background. Logically, this mechanism can work for a short depth of 400 μm. To quantify the mineral loss, the device was improvised with a CCD camera and computerization. The fluorescence loss from the lesion was subtracted from the fluorescence of the tooth, quantifying the mean and maximum fluorescence loss from the lesion as well as the area of the lesion in square mm.[47] A further modification is a portable device that uses a noncoherent blue light from a 50 W xenon arc lamp emitting blue light at 370 nm.[48]

The electrical conductivity of a normal tooth is less compared to a demineralized lesion. The porous enamel after demineralization is filled with an ionic fluid which increases the electrical conductivity. Alternately, the electrical resistance or impedance is reduced in the porous enamel. The device that measures electrical conductivity is the electrical caries meter (ECM) and the electrical impedance is measured with electrical impedance spectroscopy (EIS). The ECM is a fixed frequency device that is used in a site-specific manner or a surface-specific manner. The latter is used in epidemiological surveys. The ECM displays quantitative data that enables monitoring of lesion progression. The diagnostic results are more sensitive, but less specific than the visual aid.[49]

Summarizing, the lack of sensitivity in a gross visual examination has been the sole impetus for the evolution of a long trail of diagnostic aids. Systematic reviews[50] show that though the sensitivity has been improved with technology by lowering the diagnostic threshold, the specificity of visual examination still remains consistently unsurpassed[27] [Table 3]. The novel aids have not been revolutionary enough to replace the traditional methods, in terms of cost effectiveness, easy clinical usage, and improved patient health benefits. Currently, clinical visual examination reigns as the prime aid in the detection of caries, as is evident in the emergence of ICDAS. Nevertheless, the other aids can be used to complement the visual aid.

Thus the evolutionary cycle in lesion detection has culminated where it started, namely, the visual–tactile examination, but a very sophisticated one with stringent diagnostic criteria and with technological gadgets to assist.

**Table 3:** Sensitivity and specificity of various diagnostic aids for caries lesion detection[27,31,34,44,45,46,50,62]

	Sensitivity	Specificity
Visual inspection		
For cavitated occlusal lesions	0.62	0.93
For noncavitated occlusal lesion	0.12	0.93
For approximal cavitated lesion	0.12–0.50	0.99
Visual inspection + probe		
For noncavitated occlusal lesion in dentin	0.14	0.93
For cavitated occlusal lesion in dentin	0.82	0.93
For approximal cavitated lesion	0.12–0.50	0.99
Visual inspection in ICDAS		
For enamel and dentinal lesions	0.73	0.91
For dentinal lesions only	0.83	0.94
Bite-wing radiograph		
For noncavitated fissure caries	0.45	0.83
For cavitated occlusal lesion in dentin	0.79	0.83
For approximal enamel lesion	0.29	
For approximal dentinal lesion	0.23	1.00
Direct digital radiography		
For occlusal enamel lesions	0.24–0.31	0.72–0.80
For proximal enamel lesions	0.35	0.80
For occlusal dentinal lesions	0.21–0.69	0.84–0.97
For proximal dentinal lesions	0.16–0.52	0.95–0.96
FOTI		
For occlusal surface	0.13	0.99
For approximal enamel lesion	0.19	
For approximal dentinal lesion	0.68	0.99
DIFOTI		
For smooth surface lesions	0.43	0.87
For occlusal lesions	0.67	0.87
For approximal lesions	0.56	0.76
Diagnodent		
For occlusal dentinal caries	0.76	0.87
For occlusal enamel caries	0.42	0.95
ECM		
For enamel caries	0.70–0.92	0.78–1.00
For dentinal caries	0.39–0.97	0.56–0.98

### Examination of caries lesions

**Figure 4:**
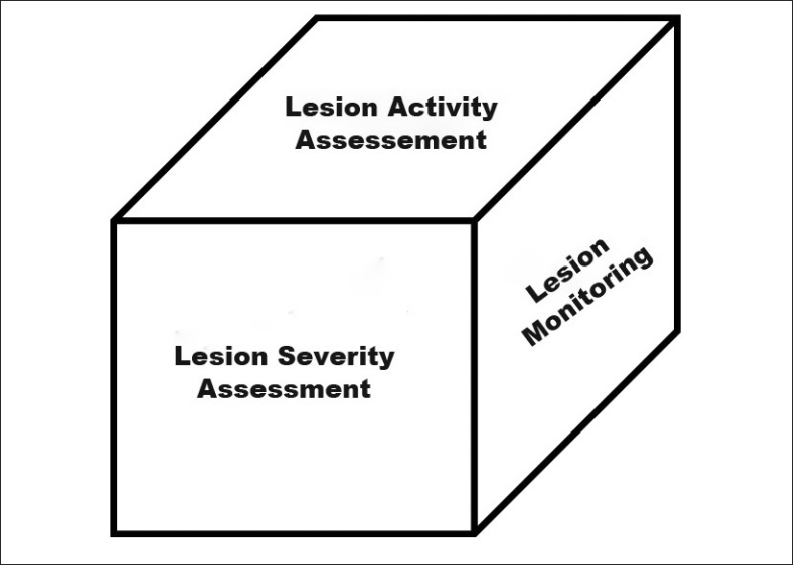
Illustration depicting a ‘Caries cube’, showing the three assessment parameters[52]

The statement lesions are detected on extracted teeth[51] is explicit enough to emphasize that the caries diagnosis and detection goes further than just that. Once the lesion is detected, it has to be examined for the following parameters: lesion extent, lesion activity, and lesion behavior over time[52] [Figure 4].

A noncavitated white spot lesion detected on a smooth surface may be an ongoing demineralization at the point of detection, or it may be an arrested demineralization, or it may be a scar tissue remnant after remineralization. Any of these three situations is very much possible keeping in mind the dynamic disease process. The same dynamism may also result in the transformation of these lesions for the betterment or for the worse. A regressed lesion can become an active demineralization or an active demineralization can transform into an arrested lesion. The status of the lesion at a point of time and its progression over a period of time assume tremendous significance, since the vital treatment decision process rests on them. For instance, an active, noncavitated white spot lesion in a high-risk individual has the maximum probability of aggressively progressing. The treatment then becomes rigorous and follow-up regime/monitoring becomes mandatory.

The progression or regression of the lesion is directly associated with the caries disease risk status. The risk status of the disease too can shift from high risk to low risk or the vice-versa, a probability that is related to many patient-related factors, such as the dental awareness, socioeconomic status, psychosocial factors, dietary habits, and oral hygiene status. Caries monitoring implies not only the observation of a change in the lesion status, but change in the risk status too, over a period.

## DENTAL CARIES PROGNOSIS – A GATEWAY TO MANAGEMENT

Both the assessment of caries activity status and the assessment of caries risk prediction help to determine the prognosis of the dental caries, a mission that is as significant as diagnosis and detection, for doing the right thing, done right, at the right time for the right person.[53]

### CRA: The prognostic aspect

The past caries experience of the patient and the socioeconomic status of the patient have been proposed as strong predictors in caries risk assessment.[54,55] The biological factors that have been elaborated in the diagnostic aspect act as predictors as well.

The data related to caries prediction are obtained from a detailed history and clinical examination. Comprehensive dialoguing with the patient provides clues on past dental treatments, systemic illnesses predisposing to caries, socioeconomic status, and familial tendency. A detailed history can also reveal the patient's oral hygiene habits, life-style habits (e.g., smoking/consumption of alcohol) and dietary habits, and fluoride exposure (exposure to fluoridated water/fluoridated tooth paste/mouth wash), all of which can have a definite impact on the future of caries [Table 4].

The presence of restorations and edentulous spaces should be corroborated with the past dental history, if they are the effect of a carious or noncarious disfigurement. The frequency of the past treatment directly implies the frequency of a new lesion occurrence. Systemic illnesses can alter the salivary parameters, diet, attitude, and psychomotor skills and hence can affect the caries incidence and progress. Social history conveys the economical, educational, and professional status of the patient, which also conveys the attitude of the patient toward oral health and dental awareness. Family history revealing caries in siblings indicates a strong familial tendency. The presence of multiple active lesions, especially in the anterior teeth or in proximal surfaces along with a poor oral hygiene, collectively forms vital clinical parameters for caries prediction.[56]

**Table 4:** Predictor factors of caries risk assessment indicating high risk

		
Personal history	Susceptible age factor
	(very young/very old/teen age)
Past dental history	Premature extractions
	Multiple restorations
	History of frequent restorations
Medical history	Medically compromised
	Handicapped
	Xerostomia
	Cariogenic medication
Social history	Socially deprived
	Low knowledge of dental diseases
	Irregular attender
	Low dental aspirations
	Living in fluoridated area
Familial history	High caries in siblings
Oral hygiene habits	Use of fluoridated tooth paste and brush
	Regular brushing (twice a day)
	Fluoridated mouth wash
Life-style habits	Smoking
	Alcohol consumption
	Irregular working hours/shift system
	Irregular eating habits
	Ready access to snacks
Clinical evidence	New lesions
	Premature extractions
	Anterior caries/restorations
	Multiple restorations
	No fissure sealants
	Orthodontic treatment
	Partial dentures

Modified[15]

### Caries lesion activity assessment

Caries activity is “the summation of the dynamics of the caries process resulting in the net loss, over time, of mineral from a caries lesion – i.e., there is an active lesion progression.” An active caries is a “lesion, from which, over a specified period of time, there is net mineral loss, i.e., the lesion is progressing.” An inactive caries is a “lesion which is not undergoing net mineral loss – i.e., the caries process in a specific lesion is no longer progressing.”[57] These are recorded so, using certain characteristic changes in the lesion appearance, at the time of detection or at two or more points of time (monitoring). This concept implies that not all demineralized white spots inevitably result in progressive destruction. Especially with the exposure of fluoride, a reversal of an ongoing demineralization is always a promising possibility. A simple removal of plaque from a white spot lesion can also contain the progress. This is surprisingly not a new concept as is evident from the visionary works of GV Black documented in the early 1900s.[58] However, the absence of reliable criteria had been the deterrent factor in its popular usage. The subsequently available literature is also meager, but is emerging with appreciable reliability and validity [Table 5a and b].

The surface characteristics, such as color and texture have been adopted as defining criteria. The pathoanatomical changes in the enamel and dentin have been clearly delineated to aid in such a registration [Table 6]. As the presence of biofilm is one of the strong influencing factors for active demineralization, plaque stagnation and plaque stagnation areas have also been included in the assessment model.[4,44,58,59,60] Recently, scoring of the active and inactive lesions has been proposed using three parameters: (a) the lesion severity assessment based on ICDAS II and rearranging the lesions according to the color, such as brown/white/microcavitated/shadowed/frank cavitation; (b) lesion location based on the plaque stagnation area; and (c) tactile feeling with ball-ended probe, such as smooth/rough.[61]

**Table 5a:** Nyvad's caries diagnostic and activity assessment criteria[38]

Score	Category	Criteria
0	Sound	Normal enamel translucency and texture (slight staining allowed in otherwise sound fissure)
1	Active caries (intact surface)	Surface of enamel is whitish/yellowish/opaque with loss of lustre; feels rough when the tip of the probe is moved gently across the surface; generally covered with plaque. No clinically detectable loss of substance Smooth surface: caries lesion typically located close to the gingival margin Fissure/pit: Intact fissure morphology; lesion extending along the walls of the fissure
2	Active caries (surface discontinuity)	Same criteria as score 1 Localized surface defect (microcavity) in enamel only No undermined enamel or softened floor detectable with the explorer
3	Active caries (cavity)	Enamel/dentine cavity easily visible with the naked eye; surface of cavity feels soft or leathery on gentle probing. There may or may not be pulpal involvement
4	Inactive caries (intact surface)	Surface of enamel is whitish, brownish, or black. Enamel may be shiny and feels hard and smooth when the tip of the probe is moved gently across the surface. No clinically detectable loss of substance Smooth surface: caries lesion typically located at some distance from the gingival margin. Fissure/pit: intact fissure morphology; lesion extending along the walls of the fissure
5	Inactive caries (surface discontinuity)	Same criteria as score 4 Localized surface defect (microcavity) in enamel only No undermined enamel or softened floor detectable with the explorer
6	Inactive caries (cavity)	Enamel/dentin cavity easily visible with the naked eye; surface of cavity may be shiny and feels hard on probing with gentle pressure No pulpal involvement
7	Filling (sound surface)	Same as score 0
8	Filling + active caries	Caries lesion may be cavitated or noncavitated
9	Filling + inactive caries	Caries lesion may be cavitated or noncavitated

**Table 5b:** Ekstrand's caries diagnostic and activity assessment criteria

Detection criteria		Activity criteria	

Code	Criteria	Code	Criteria
0	No or slight change in enamel translucency after prolonged air-drying (>5 s)	0	No or slight change in enamel translucency after prolonged airdrying (>5 s)
1	Opacity or discoloration hardly visible on the wet surface, but distinctly visible after air-drying	1	Opacity (white) hardly visible on the wet surface, but distinctly visible after air-drying
		1a	Opacity (brown) hardly visible on the wet surface, but distinctly visible after air-drying
2	Opacity or discoloration distinctly visible without air-drying	2	Opacity (white) distinctly visible without air-drying Opacity (brown) distinctly visible
		2a	without air-drying
3	Localized enamel break-down in opaque or discolored enamel and/or grayish discoloration from the underlying dentine	3	Localized enamel break-down in opaque or discolored enamel and/or grayish discoloration from the underlying dentine
4	Cavitation in opaque or discolored enamel exposing the dentine	4	Cavitation in opaque or discolored enamel exposing the dentine

Modified[60]

### Toward ultimatum

The long process of diagnosis of the disease, and the detection and prognosis of dental caries culminate in a single point of focus, i.e., treatment. The treatment is not just to target the lesions alone, as practiced since antiquity, but to manage the patient in a holistic way, to ensure the best oral health for a long time. Though preventive measures constitute a major part of the management model, given the status that the biofilm is ubiquitous in the mouth and caries is life-style-influenced disease, it remains to be answered if it can be prevented in a true sense or just controlled[6] over life-time. The eradication of dental caries disease seems to be a far-fetched dream, in the same league. The subsequent section shall attempt to answer this.

**Table 6:** The pathoanatomical features of the enamel and dentin in active and arrested lesions[60]

	Visual	Tactile
Enamel		
Active	The lesion is whitish/yellowish; the lesion is chalky (lack of luster); the lesion can be cavitated or not	The lesion feels rough to probing; probing might or might not find cavity
Arrested	The lesion is more yellowish/brownish than whitish; the lesion is more shiny than matte; the lesion can be cavitated or not	The lesion feels more smooth than rough; probing might or might not find a cavity
Coronal dentine		
Active	The lesion may manifest itself but demineralized enamel; if a cavity extends into the dentine, the dentine appears yellowish/brownish	Dentine soft to probing
Arrested	The lesion may manifest itself as a shadow below the intact but demineralized enamel; if a cavity extends into the dentine, the dentine appears brownish	Harder than at the active lesion but not as hard as sound dentine
Root dentine		
Active	Yellowish/brownish	Soft/leathery
Arrested	Brownish/blackish	Harder but not as hard as sound root dentine
